# Modeling of *Salmonella enterica* in the pulp and on the outer rind of Tommy Atkins mango (*Mangifera indica*) and transfer during sanitization of fruit

**DOI:** 10.1007/s42770-026-01908-w

**Published:** 2026-03-26

**Authors:** Mírian Pereira da Silva, Jacqueline Valle de Bairros, Wilmer Edgard Luera Peña

**Affiliations:** Department of Food Technology, Federal University of Viçosa, Viçosa, Minas Gerais 36570 900 Brazil

**Keywords:** Predictive model, Microbial transfer, Hydrothermal treatment, Cross-contaminaton Fruit matrix

## Abstract

Outbreaks involving *Salmonella* in mangoes from Brazil have been reported in importing countries, causing social and economic losses, especially to consumers. This study aimed to develop predictive models for the growth kinetics of *Salmonella enterica* serovar Typhi in the peel and pulp of Tommy Atkins mangoes as a function of temperature, as well as to evaluate the transfer of *S.* enterica serovar Typhimurium from contaminated to non-contaminated fruits. No significant differences in kinetic parameters were observed between peel and pulp. At lower temperatures (7, 10, and 15 °C), significant differences (*p* ≤ 0.05) were found in lag phase (λ), whereas at higher temperatures (25, 30, and 35 °C), significant differences were observed in the maximum specific growth rate (µ). The secondary models developed (R² > 0.88) adequately described the effect of temperature on λ and µ in both peel and pulp. In the transfer assay, the passage of *S.* Typhimurium from the surface of inoculated mangoes to non-inoculated fruits was low. The hydrothermal treatment with chlorine was more effective in reducing *S.* Typhimurium transfer rates compared with hydrothermal treatment without sanitizer. The results demonstrate that both the peel and pulp of mangoes support *Salmonella* growth over a wide temperature range and indicate that exposure of Tommy Atkins mango surfaces to chlorinated solution during hydrothermal treatment may reduce the risk of salmonellosis.

## Introduction

There is increasing discussion among the scientific community regarding reports of foodborne outbreaks caused by the consumption of fresh fruits and vegetables [1–3]. These products are often exposed to microbial contamination during harvesting, transportation, processing and distribution [4]. In Brazil, between 2014 and 2023, approximately occurred 6,874 foodborne outbreaks, of which 1.8% were associated with the consumption of fruits, fruit products and related items. *Salmonella* was the most frequently identified pathogen, responsible for approximately 9.6% of these outbreaks [5].

In the European Union (EU), *Salmonella* is the second most common cause of zoonotic disease and is frequently associated with foodborne outbreaks. Each year, more than 91,000 cases of salmonellosis are reported in the EU, with the overall economic burden of human salmonellosis is estimated to reach up to 3 billion euros annually [6]. Meanwhile, in the United States, *Salmonella* is estimated to cause approximately 1.35 million infections, 26,500 hospitalizations, and 420 deaths each year [7].

In recent years, the consumption of fresh produce has grown significantly, driven by increased awareness of healthy dietary and the nutritional benefits of these foods. However, this increased consumption has been paralleled by a notable rise in reported foodborne outbreaks associated with fresh produce. *Salmonella* is an enteropathogenic bacteria widely disseminated in the environment and capable of contaminating fruits through contact with soil, dust, water, animal feces and human handling [8]. Moreover, the pathogen may be transferred via cross-contamination during fruit peeling and/or cutting, as well as internalization during hydrothermal treatments [9, 10].

Fruits exported to other countries must undergo different treatments required by the countries. Mangoes exported for United States must receive hydrothermal treatment, which involves immersing the fruit in hot water at 46.1 °C for 65 min in a chlorinated solution containing 200 mg/L of chlorine residual total (CRT) [11]. The efficacy of this treatment should ensure the elimination of pathogens and prevent their transfer, which may occur during the process. The occurence of outbreaks even after treatment is, in part, attributed to the fact that fruit processing sheds are often open structures, allowing the entry of animals that carry human pathogens such as *Salmonella*. This can lead to contamination of the water used during the heat treatment.

Several studies have shown the occurrence of *Salmonella* in mango [10, 12, 13], tomato [14], papaya [15], melons and watermelons [16]. The presence of this pathogen in fruits may be associated with the favorable conditions of pH, water activity, and temperature [17]. In this context, studies have recently reported the use of modeling approaches to evaluate the effect of these factors on *Salmonella* growth in fruits and vegetables [9, 18–20].

Considering the above mentioned the objective of this research was to develop a mathematical model to predict the growth of *Salmonella* on the outer rind and pulp of *Tommy Akti*ns mango as function of temperature, and to evaluate the effect of using chlorinated water during the hydrothermal treatment stage in controlling and transferring this pathogen to other uncontaminated fruits.

## Materials and methods

### Determination of growth parameters (lag phase λ and Growth rate µ) of *S. enterica* subsp. *entérica* serovar Typhi in Tommy Atkins mango peel and pulp

#### Samples

Tommy Atkins variety mangoes were used in this stage of the study. The fruits were cleaned according to the procedures described in the Minimum Processing of Fruit*s* manual [21]. The mangoes were washed in running water using a sponge and neutral detergent, with scrubbing for 10 min. Subsequently, sanitization was performed in a chlorine solution at a concentration of 200 mg L^-1^ of total residual chlorine, for 5 min, pH 6.5 ± 0.2. The chlorine solution was prepared from the commercial product (Sumaveg, Brazil) in water with a temperature between 10 and 15 °C [21]. Sodium thiosulfate (Sigma-Aldrich, USA) at 0.25% (m/v) was applied for 5 min to neutralize the sanitizing agent [18].

#### Bacterial culture

A strain of *S. enterica* subsp. *enterica* serovar Typhi (NR-074799.1) isolated from lettuce was used. An aliquot of strain, frozen at -80 °C, was transferred to Brain Heart Infusion (BHI) broth (Thermo Fisher Scientific Oxoid, Basingtoke, UK) and incubated at 35 °C ± 1 °C for 24 h. The culture was transferred again to the BHI broth following incubation at 35 °C ± 1 °C for 18 h. For standardization of the inoculum, a spectrophotometer (model Kazuaki IL-227, Kazuaki, Brazil) was used, and the reads were done at the wavelength of 625 nm, according to the 0.5 McFarland standard [18].

#### Inoculation and enumeration of *S. enterica* in mango pulp and peel

Prior to pathogen inoculation, the pH and water activity of the outer rind and pulp of Tommy mango were measured. Measurements were obtained using a pH meter (lon PHB-5000; [22]). Water activity was determined employing a 4TE Dewpoint Water Activity Meter (Aqualab Decagon, Washington, USA).

Peel samples were obtained using a sterile scalpel and a 5 cm² stainless steel mold. Mango pulp was collected with a sterile knife by cutting approximately 1 g portions. Both peel and pulp samples were individually placed in sterile Petri dishes and inoculated with 0.1 mL of *S.* Typhi to achieve a final concentration of approximately 2 log CFU/g. Samples were incubated at different temperatures (7, 10, 15, 20, 25, 30, and 35 °C), and bacteria enumeration was performed at specific time intervals according to the incubation temperature. These intervals were defined based on growth predictions generated using the Pathogen Modeling Program (PMP, version 7.0), developed by the United States Department of Agriculture – Agricultural Research Service (USDA–ARS).

For bacterial enumeration, each sample was individually diluted in 0.1% peptone water (BD, Difco, Brazil) and aliquots were plated on Xylose Lysine Deoxycholate (XLD) agar (Kasvi, Italy) using the microdroplet technique. Plates were incubated at 37 ± 1 °C for up to 24 h, and results were expressed as log CFU/g of sample. The experiment was performed twice, each in duplicate.

#### Predictive model

The Baranyi model [23] (Eqs. 1–3) was fitted to the growth data of *S.* Typhi in mango peel and pulp under storage conditions of 7 to 35 °C using DMFit, version 2.1. Excel (www.ifr.ac.uk/safety/DMFit*).*

1 $$\mathrm{l}\mathrm{n}(N\left(t\right)=\mathrm{ln}\left({N}_{0}\right)+{\mu}_{max}A\left(t\right)-ln⌊1+\frac{{e}^{{\mu}_{max}A\left(t\right)}-1}{{e}^{({N}_{max}-{N}_{0})}}⌋$$

2 $$A\left(t\right)=t+\frac{1}{{\mu}_{max}}ln\left[\frac{{e}^{(-{\mu}_{max}t)}+{q}_{0}}{1+{q}_{0}}\right]$$

3 $$\text{}\mathrm{=}\frac{ln\left[1+\frac{1}{{q}_{0}}\right]}{{\mu}_{max}}$$

Where: ln(*N(t))* [ln(UFC/mL)] is the natural logarithm of the cell concentration at time *t [h]*; ln(*N*_*0*_) is the natural logarithm of the initial cell concentration; *µ*_*max*_ *[1/h* represents the maximum specific growth rate; *A(t)* is the adjustment function; ln(*N*_*max*_) is the natural logarithm of the maximum cell concentration; *q*_*0*_ is a measure of the physiological state of the cell when *t* = *t*_*0*_ and λ represents the lag time.

Ratkowsky model [24] was used to describe the lag phase λ and the growth rate µ as a function of temperature (Eq. 4).

4 $$ln\lambda\:or\sqrt{{\mu}_{max}}=\alpha(T-T0)$$

To calculate the lag phase λ, the data were transformed into natural logarithm (ln λ). µ_max_ is the maximum specific growth rate, 𝛼 is the regression coefficient, T is the incubation temperature (ºC); T0 is the minimum temperature (ºC).

The models obtained in this study were compared with models implemented with growth data generated by the ComBase Predictor program (https://www.combase.cc/index.php/en/*)* under the pH and Aw conditions of the analyzed fruit.

### Evaluation of *S.* typhimurium transfer from contaminated to uncontaminated mangoes during hydrothermal bath

#### *Salmonella* thyphimurium inoculation

Ripe, uninjured and sanitized mangoes were used, following the procedure described in Sect.  2.1.1. Six experimental scenarios were established (Table 1), with 10 fruits per scenario, individually identified from 1 to 10. Each mango was inoculated with 3 mL of the *S.* Thyphimurium (ATCC 14028) suspension by drip application [25]. Two inoculum concentrations were studied to simulate different contamination levels: 5 log CFU/mango and 8 CFU/mango.

#### Hydrothermal treatment

In a previously sanitized stainless steel container, 20 L of a chlorinated solution containing 200 mg/L of of total residual chlorine at pH 6.5 ± 0.2 were prepared using sterilized water according to the manufacturer (Sumaveg Diversy Brasil Indústria Química Ltda, São Paulo, Brazil). The chlorinated solution was previously heated using two electric boilers (EB Distribuidora, Gravataí, RS, Brazil, 770 W power) to a temperature of 46.1 ± 1 °C to simulate the hydrothermal treatment. The temperature was measurements at 5-min time intervals.

Contaminated and uncontaminated mangoes were immersed in the heated sanitizing solution to simulate the hydrothermal treatment. The experiments in Table 1 were subjected to hydrothermal treatment for 65 min under controlled temperature conditions. There was no agitation of the mangoes during the bath.

**Table 1:** Hydrothermal treatments conducted at two inoculum concentrations (5 and 8 log CFU/mango) in six experiments (A, B, C, D, E, F) according to the number of inoculated mangoes (1, 3, 5), simulating low (10%), medium (30%) and high (50%) contamination levels

Inoculum concentration (log CFU/mango)	Experiments	Inoculated mangoes	Contamination (%)	Contamination levels
	A	1	10	low
5	B	3	30	medium
	C	5	50	high
	D	1	10	low
8	E	3	30	medium
	F	5	50	high

After the hydrothermal bath simulation, 2 L of a 0.25% (m/v) sodium thiosulfate solution (Sigma-Aldrich, São Paulo, Brazil) were added for 5 min to neutralize the sanitizing agent. Subsequently, the mangoes were removed from the stainless steel container and placed in sterilized Petri dishes at room temperature for 30 min to allow cooling.

Similarly, the procedures described for experiments A, B, C, D, E, and F, as well as the two inoculum concentrations analyzed (5 and 8 CFU/mango), were also conducted without the addition of chlorine to the hydrothermal treatment water. The transfer of *S.* Thyphimurium from contaminated to uncontaminated mangoes was verified in accordance with item 2.1.3.

### Data analysis

The kinetic growth parameters of *S.* Typhi (lag phase (λ) and growth rate (µ)) on mango peel and pulp were evaluated using analysis of variance (ANOVA) [26], followed by Tukey’s test. Statistical analyses were performed using the Statistical Analysis System software (SAS Institute, Cary, NC, USA), version 9.1. Results from the *S.* Tiphymurium transfer assays on mango surfaces were assessed by descriptive analysis.

## Results and discussion

### Determination of growth parameters of *S. enterica* in Tommy Atkins mango peel and pulp

*S.* Typhi grew in all temperatures studied (7º to 35 °C), both on the outer rind and in mango pulp, situation reported in several studies [12, 27]. The presence and growth of *Salmonella* on fruit peels may facilitate its transfer to the pulp, leading to potential proliferation [31, 32]. Since these products are often consumed raw, their microbiological safety represents a significant public health concern.

**Fig. 1:**
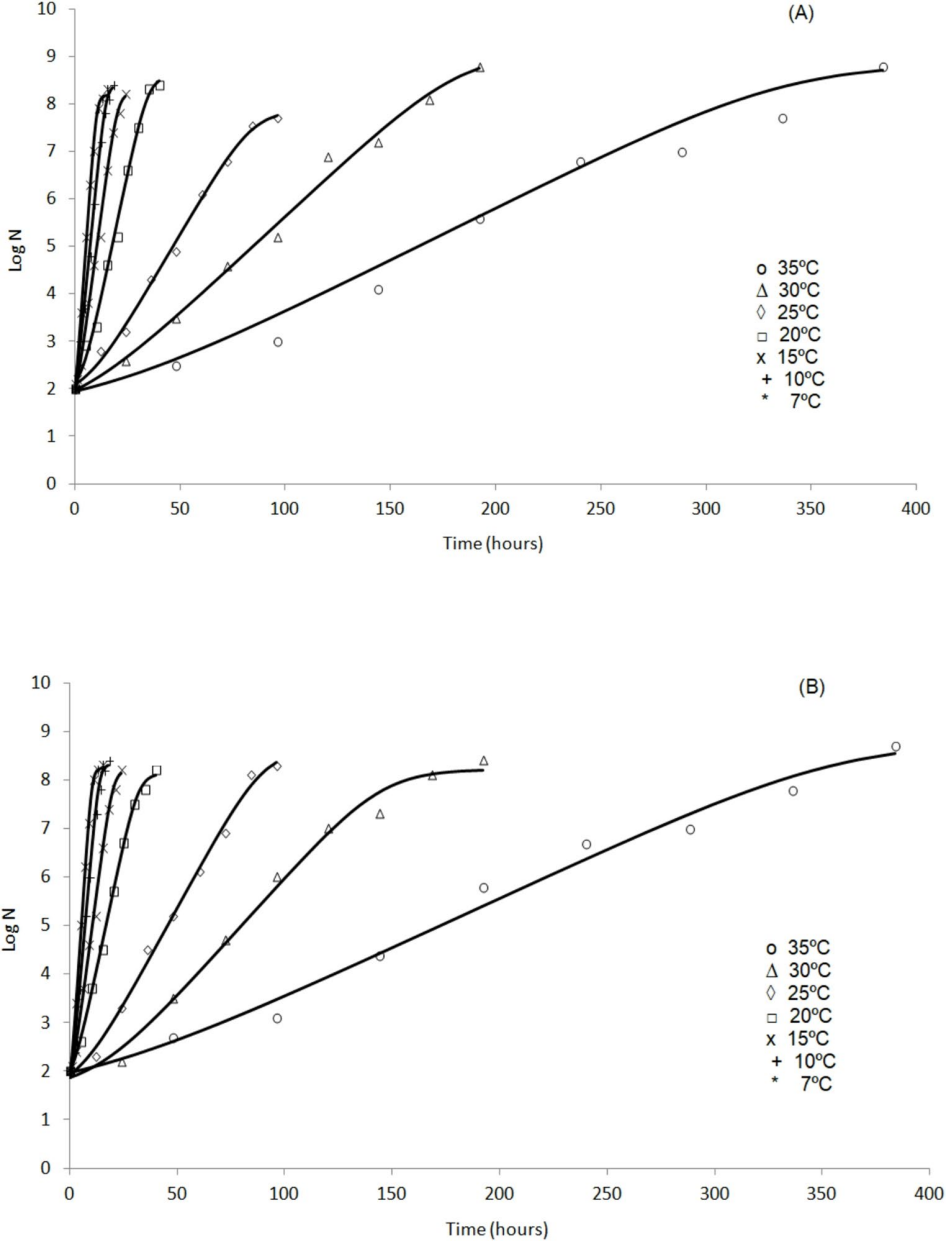
The Baranyi and Roberts model adjusted to the growth data of *S.* Typhi in mango: (**A**) peel and (**B**) pulp

Table 2 presents the kinetic growth parameters of *S.* Typhi on mango peel and pulp, estimated by the Baranyi and Roberts model [23], with R^2^ > 0.98. Temperature affected the kinetic parameters. Increasing temperature resulted in a reduction in lag phase duration (λ) and an increase in the growth rate (µ), with optimal growth conditions observed at 35 °C. This temperature-dependent behavior is consistent with the Arrhenius principle and with the reported optimal growth range for *Salmonella* spp [24, 30, 31]. Therefore, the commercialization of these fruits becomes a matter of concern, given that these fruits are sold without proper refrigeration, which may favor the proliferation of foodborne pathogens such as *Salmonella*. Figure 1 shows that the Baranyi and Roberts model fitted well to the bacterial growth data.

**Table 2:** Growth kinetic parameters (lag time (λ) and growth rate (µ)) of *S.* Typhi in mango peel and pulp under storage conditions at 7, 10, 15, 20, 25, 30 and 35 °C

Temperature (ºC)	Kinetic Parameters	Peel	Pulp
7	*λ* (h) *µ* (1/h)	25.32 ± 3.07^aC^ 0.020 ± 0.001^aA^	25.21 ± 2.78^aC^ 0.021 ± 0.001^aA^
10	*λ* (h) *µ* (1/h)	11.60 ± 3.52^aB^ 0.0413 ± 0.030^aA^	16.38 ± 4.00^aB^ 0.0521 ± 0.011^aA^
15	*λ* (h) *µ* (1/h)	8.34 ± 1.27^aAB^ 0.083 ± 0.011^aAB^	7.67 ± 1.15^aA^ 0.081 ± 0.014^aA^
20	*λ* (h) *µ* (1/h)	3.01 ± 0.13^aA^ 0.204 ± 0.001^aB^	2.48 ± 0.80^aA^ 0.21 ± 0.013^aC^
25	*λ* (h) *µ* (1/h)	1.00 ± 0.71^aA^ 0.362 ± 0.030^aC^	1.53 ± 0.52^aA^ 0.371 ± 0.044^aD^
30	*λ* (h) *µ* (1/h)	0.66 ± 0.26^aA^ 0.510 ± 0.080^aD^	1.48 ± 0.36^aA^ 0.612 ± 0.050^aB^
35	*λ* (h) *µ* (1/h)	0.50 ± 0.36^aA^ 0.692 ± 0.010^aE^	0.69 ± 0.31^aA^ 0.630 ± 0.021^aB^

^a^ Different lowercase letters in the same line and for the same kinetic parameter indicate significant differences (p ≤ 0.05) according to ANOVA followed by Tukey's t-test, as a function of the storage temperature of S. Typhi between the food matrix (mango peel or pulp).ABCDE Different capital letters in the same column and for the same kinetic parameter indicate significant differences (p ≤ 0.05) according to ANOVA followed by Tukey's t-test, as a function of the storage temperature of S. Typhi per food matrix (mango peel or pulp). Results expressed as mean and standard deviation of two replicates

No significant differences were observed in the kinetic parameters λ and µ of *S.* Typhi between mango peel and pulp at the same temperature (*p* > 0.05). However, previous studies indicate that these kinetic parameters can be influenced by the fruit matrix, as differences in surface characteristics, nutrient availability, and physicochemical properties may influence microbial behavior [32, 33]. Nevertheless, temperature had a significant effect on both parameters (*p* ≤ 0.05). Lower temperatures were associated with extended lag phases, reflecting reduced metabolic activity and stress responses triggered by exposure to cold [34–36]. As the temperature decreased, the bacterial adaptation period increased, as shown in Table 2.

The survival and growth capacity of the pathogen is influenced by its physiological state and environmental conditions ^35^. Under refrigeration, *Salmonella* can activate adaptive stress responses that enhance persistence at low temperatures [36], which may explain the elevated λ observed at 7 °C.

The Tommy Atkins mango variety has a reported pH range of 3.5 to 3.7 [37] reaching approximately 4.29 at full ripeness [38]. The ability of *S.* Typhi to grow in mango pulp is supported by the intrinsic properties of the fruit. The measured pulp pH (4.2) and water activity (0.987) were within the established growth limits for *Salmonella* [17, 39–41], supporting the observed proliferation in mango pulp. Under all experimental conditions, maximum populations between 8 and 9 log CFU/g were reached in both peel and pulp, corroborating previous observations in other fruits matrices [27]. In the present study, mango pulp acidity did not prevent the pathogen from reaching high population levels. It is possible that the acid stress caused by the mango pulp pH may have activated the acid tolerance response system in *Salmonella*, facilitating survival and multiplication under these conditions [42, 43].

These findings showed that *Salmonella* growth can occur on the peel and in the pulp of various fruits, and once multiplication begins, high bacterial populations may be found. Therefore, storing fruits at temperatures between 10 and 13 °C [48, 49] may help to control the development of this pathogen. In this context, it becomes evident that the temperature to which mangoes are exposed after harvest is a critical factor influencing the behavior of *S.* Typhi and must be controlled during transportation, storage, distribution, and retail, until it reaches the final consumer. In the present study, to evaluate the temperature dependence of lag phase duration and growth rate, the square root model [24] was applied. For model implementation, the mean values of λ and µ for *S.* Typhi in mango peel and pulp were used and compared with with predictions generated using ComBase database, as shown in Table 3; Fig. 1.

**Table 3:** Secondary models for lag phase λ and growth rate µ of *S.* Typhi in mango peel and pulp and obtained with data generated by the ComBase program

Matrix	Model of lag time	*R* ^2^	Model of growth rate	*R* ^2^
Outer rind	$Ln\lambda=-0.1459(T-27.91)$	0,9697	$\sqrt{\mu}=0.0025(T-2.29)$	0.9997
Pulp	$Ln\lambda=-0.1279(T-30.80)$	0,9557	$\sqrt{\mu}=0.0266(T-2.19)$	0.9436
Combase	$Ln\lambda=0.0183(T+140.77)$	0,9673	$\sqrt{\mu}=0.0213(T-4.60)$	0.9979

According to Table 3, the R^2^ values of the secondary models for lag time and growth rate of *S.* Typhi in mango peel and pulp were > 0.94, indicating a good fit of the model. Similarly, models generated using growth data obtained through ComBase Predictor also showed high R² values.

**Fig. 2:**
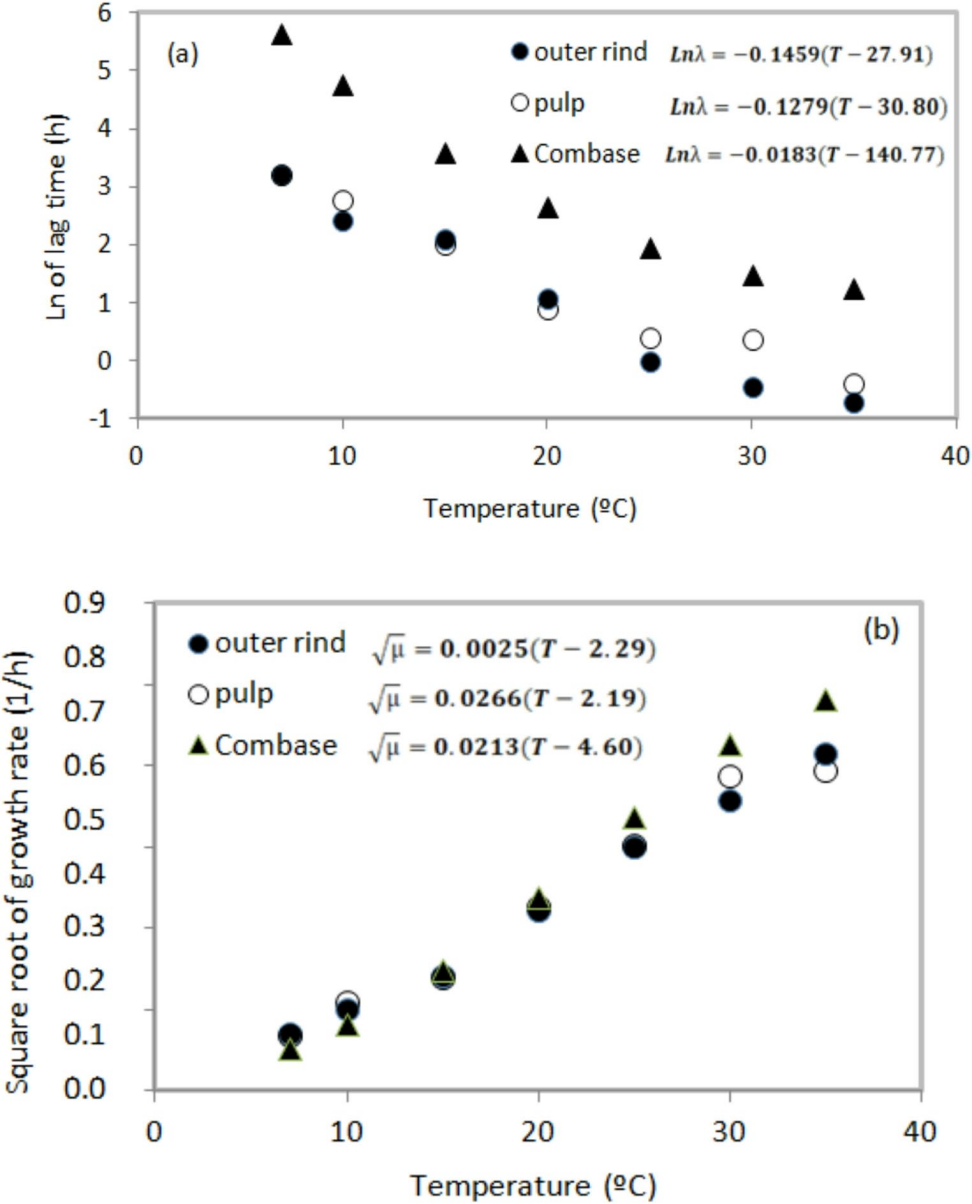
Model for lag time of *S.* Typhi inoculated on outer rind (●) and pulp (o) of mango and ComBase (▲) (**A**) and rate square (**B**)

As observed in Fig. 2, temperature affected ln λ and √µ values of *S.* Typhi, with differences between mango matrices (peel and pulp) ComBase predictions. At 10 °C, the ln λ value predicted by ComBase was 1.98 h higher than that obtained for mango pulp. ComBase predictions were similar to experimental values at lower temperatures (7–10 °C), whereas at temperatures above 20 °C the model overestimated √µ compared to mango peel and pulp, with the largest difference (0.10 1/h) observed at 35 °C. These observed differences may result from nature of the data used to generate growth curves. Computational models such as ComBase, are predominantly based on data obtained from experiments using culture media [46], whereas the models developed in this study were based on a food matrix. Therefore, the secondary models developed here (Table 3) allow more accurate prediction of *S.* Typhi growth in Tommy Atkins mango peel and pulp across a wide temperature range.

### Cross-contamination of *S.* thyphimurium during hydrothermal bathing of mangoes

In this study, the experiments simulated the potential transfer of *S.* Thyphimurium from contaminated to uncontaminated Tommy Atkins mangoes during hydrothermal treatment. Cross-contamination was influenced by the initial inoculum level, the number of contaminated fruits, and the presence of chlorine in the treatment water (Table 4).

**Table 4:** Occurrence of *S.* Thyphimurium on mango surfaces after the hydrothermal treatment step with and without chlorine addition

Inoculum concentration (log CFU/mango)	Inoculated mangoes	Experiments	Hydrothermal treatment with chlorine (%)	Hydrothermal treatment without chlorine (%)
	1	A	ND	10
5	1, 2 e 3	B	ND	30
	1, 2, 3, 4 e 5	C	20	70
	1	D	10	20
8	1, 2 e 3	E	40	50
	1, 2, 3, 4 e 5	F	70	80

ND: Not detected

The bacteria were not detected in only two experiments (A and B) using an inoculum Strawnlevel of 5 log CFU/mango. In these treatments, the combination of chlorine and low initial contamination levels may have contributed to the efficiency of hydrothermal treatment. The hydrothermal bath applied in this study is recommended by the United States Department of Agriculture [11].

The effectiveness of hydrothermal treatment at 46.1 °C for 65 min, combined with 200 mg/L of chlorine ^11^ in controlling *Salmonella* transfer on mango surfaces under operational conditions recommended for food safety, has not been previously reported in the scientific literature. Similar studies have shown that temperature, chlorine concentration, and fruit peels to water temperature ratio influence pathogen reduction on fruit surfaces [47–49], and the combined effect of these parameters on *Salmonella* transfer during mango hydrothermal treatment has been reported.

The role of process water as a vehicle for pathogen transfer during fruit processing is well established [50, 51]. Water may become contaminated for distinct reasons, including exposure to animals in processing facilities. During post-hydrothermal treatment, the dietary fibers of the heated fruit contract during cooling and an internal hydrostatic pressure difference between the gases absorbs water, as well as pathogens [52]. Once internalized, surface sanitation becomes ineffective in eliminating the pathogen [27]. Therefore, the water used in fruit processing must be properly filtered and chlorinated. Moreover, if cold water is applied after the hot water treatment, a minimum cooling period of 30 min should be observed between the hot and cold water steps [11].

Once the pathogen is internalized, conventional treatments to decontaminate the fruit surface will not reduce the potential risk of a foodborne outbreak when the product is consumed [30]. The use of untreated, inadequately chlorinated or secondarily contaminated water during processing may function as a vector for mango contamination and pathogens internalization. This process has been previously documented in tomatoes [53], apples [52] and mangoes [10, 27] with the authors concluded that internalization frequently occurs through the peduncle [11]. However, *Salmonella* transfer to the pulp during peeling and cutting has also been demonstrated [54–56].

Overall, mangoes treated with chlorinated water showed lower *Salmonella* transfer rates compared with treatments without sanitizer (Table 4). Increasing water temperature or chlorine concentration beyond recommended limits is not a practical alternative, as it may compromise fruit quality and sensory attributes [57, 58]. Therefore, maintaining appropriate chlorination during hydrothermal treatment represents an effective strategy to reduce the risk of *Salmonella* cross-contamination in Tommy Atkins mangoes.

## Conclusion

The results confirmed that *S.* Typhi was able to adapt, grow and reach high microbial populations in both mango peel and pulp across a wide temperature range. Furthermore, the secondary models adjusted in this study showed good fit to the experimental data and can therefore be applied to predict the behavior of *S.* Typhi on mango peel and pulp as a function of temperature.

The hydrothermal treatment combined with chlorine proved effective in scenarios with a low number of contaminated fruits, demonstrating an effect against the transfer of *S.* Thyphimurium to the surfaces of non-inoculated mangoes.

## References

[1] Aiyedun SO, Onarinde BA, Swainson M, Dixon RA (2021) Foodborne outbreaks of microbial infection from fresh produce in Europe and North America: a systematic review of data from this millennium. Int J Food Sci Technol 56(5):2215–2223. 10.1111/IJFS.14884

[2] Carstens CK, Salazar JK, Darkoh C (2019) Multistate Outbreaks of Foodborne Illness in the United States Associated With Fresh Produce From 2010 to 2017. Front Microbiol 10:492987. 10.3389/FMICB.2019.02667/BIBTEX 10.3389/fmicb.2019.02667 PMC6883221 31824454

[3] Denis N, Zhang H, Leroux A, Trudel R, Bietlot H (2016) Prevalence and trends of bacterial contamination in fresh fruits and vegetables sold at retail in Canada. Food Control 67:225–234. 10.1016/J.FOODCONT.2016.02.047

[4] Alegre I, Abadias M, Anguera M, Usall J, Viñas I (2010) Fate of *Escherichia coli* O157:H7, *Salmonella* and *Listeria innocua on minima* lly-processed peaches under different storage conditions. Food Microbiol 27(7):862–868. 10.1016/J.FM.2010.05.008 20688227 10.1016/j.fm.2010.05.008

[5] Brasil (2024) Surtos de doenças de transmissão hídrica e alimentar no Brasil. Ministério da Saúde. Secretaria de Vigilância em Saúde – SVS. https://www.gov.br/saude/pt-br/assuntos/saude-de-aa-z/d/dtha/publicacoes/surtos-dedoencas-de-transmissao-hidrica-e-alimentar-no-brasilinforme-2024/view. Published online 2024

[6] European Food Safety Authority (EFSA) (2025) *Salmonella* is a bacteria that cause an ikkness called salmonellosis in humans. This is a zoonoticdisease, with means it cab be transmitted directly or indirectly between animals and humans. https://www.efsa.europa.eu/en/topics/topic/salmonella. Published online 2025

[7] Centers for Disease Control and Prevention (CDC) (2023) About *Salmonella* Infection Multistate. https://www.cdc.gov/salmonella/about/index.html. Published online 2023

[8] Thomas GA, Paradell Gil T, Müller CT, Rogers HJ, Berger CN (2024) From field to plate: How do bacterial enteric pathogens interact with ready-to-eat fruit and vegetables, causing disease outbreaks? Food Microbiol 117:104389. 10.1016/J.FM.2023.104389 37919001 10.1016/j.fm.2023.104389 PMC7619211

[9] Jung J, Schaffner DW (2022) *Enterobacter aerogenes* B199A May Be an Effective Surrogate for Quantifying Transfer of *Salmonella* Newport 96E01152C-TX from Cucumber Peel to Edible Flesh and Peeler during Peeling. J Food Prot 85(10):1452–1457. 10.4315/JFP-22-110 35880908 10.4315/JFP-22-110

[10] Bordini MEB, Asturiano Ristori C, Jakabi M, Gelli DS (2007) Incidence, internalization and behavior of *Salmonella* in mangoes, var. Tommy Atkins Food Control 18(8):1002–1007. 10.1016/J.FOODCONT.2006.06.003

[11] United States Department Of Agriculture (USDA) (2016) Nonchemical treatments: heat, hot water immersion treatment. Treatment Manual. USDA-APHIS., Washington, United States

[12] Penteado AL, de Castro MFPM, Rezende ACB (2014) *Salmonella enterica* serovar Enteritidis and *Listeria monocytogenes*, in mango (*Mangifera indica* L.) pulp: Growth, survival and cross-contamination. J Sci Food Agric 94(13):2746–2751 doi:10.1002/JSFA.6619;PAGEGROUP:STRING:PUBLICATION 25328926 10.1002/jsfa.6619

[13] Godínez-Oviedo A, Cabrera-Díaz E, Palacios-Marmolejo A et al (2022) Detection, quantification, and characterization of *Salmonella enterica* in mango, tomato, and raw chicken purchased in the central region of Mexico. J Food Sci 87(1):370–382. 10.1111/1750-3841.16003 34954835 10.1111/1750-3841.16003

[14] Johnston L (2023) *Salmonella* and tomatoes. The Produce Contamination Problem: Causes and Solutions, Third Edition. Published online January 1, 2023:163–193. 10.1016/B978-0-12-819524-6.00002-1

[15] Whitney BM, McClure M, Hassan R et al (2021) A Series of Papaya-Associated *Salmonella* Illness Outbreak Investigations in 2017 and 2019: A Focus on Traceback, Laboratory, and Collaborative Efforts. J Food Prot 84(11):2002–2019. 10.4315/JFP-21-082 34265065 10.4315/JFP-21-082

[16] Andreoletti O, Lau Baggesen D, Bolton D et al (2014) Scientific Opinion on the risk posed by pathogens in food of non-animal origin. Part 2 (*Salmonella* in melons). EFSA J 12(10):3831. 10.2903/J.EFSA.2014.3831 10.2903/j.efsa.2014.3831 PMC13136943 42087975

[17] Keerthirathne TP, Ross K, Fallowfield H, Whiley H (2016) A Review of Temperature, pH, and Other Factors that Influence the Survival of *Salmonella* in Mayonnaise and Other Raw Egg Products. Pathogens 5(4):63. 10.3390/PATHOGENS5040063 27869756 10.3390/pathogens5040063 PMC5198163

[18] Scolforo CZ, Bairros JV, Rezende ACB et al (2017) Modeling the fate of *Listeria monocytogenes* and *Salmonella enterica* in the pulp and on the outer rind of Canary melons (*Cucumis melo* (Indorus Group)). LWT 77:290–297. 10.1016/J.LWT.2016.11.059

[19] Lee S, Han A, Yoon JH, Lee SY (2022) Growth evaluation of *Escherichia coli* O157:H7, *Salmonella* Typhimurium, and *Listeria monocytogenes* in fresh fruit and vegetable juices via predictive modeling. LWT 162:113485. 10.1016/J.LWT.2022.113485

[20] Son S, Bin, Lee HK, Kim SJ, Yoon KS (2024) Modeling Behavior of *Salmonella* spp. and *Listeria monocytogenes* in Raw and Processed Vegetables. Foods 13(18):2972. 10.3390/FOODS13182972 39335900 10.3390/foods13182972 PMC11430865

[21] Bastos MDSR (2006) Processamento Mínimo de Frutas. Coleção Agroindústria Familiar Brasília, DF, Embrapa Informação Tecnológica. Published online 2006:38

[22] Instituto Adolfo Lutz (2008) Métodos físico-químicos para análise de alimentos /coordenadores Odair Zenebon, Neus Sadocco Pascuet e Paulo Tiglea --. Instituto Adolfo Lutz, São Paulo. Published online 2008

[23] Baranyi J, Roberts TA (1994) A dynamic approach to predicting bacterial growth in food. Int J Food Microbiol 23(3–4):277–294. 10.1016/0168-1605(94)90157-0 7873331 10.1016/0168-1605(94)90157-0

[24] Ratkowsky DA, Olley J, McMeekin TA, Ball A (1982) Relationship between temperature and growth rate of bacterial cultures. J Bacteriol 149(1):1–5. 10.1128/JB.149.1.1-5.1982 7054139 10.1128/jb.149.1.1-5.1982 PMC216584

[25] Harris LJ, Beuchat LR, Kajs TM, Ward TE, Taylor CH (2001) Efficacy and Reproducibility of a Produce Wash in Killing *Salmonella* on the Surface of Tomatoes Assessed with a Proposed Standard Method for Produce Sanitizers. J Food Prot 64(10):1477–1482. 10.4315/0362-028X-64.10.1477 11601693 10.4315/0362-028x-64.10.1477

[26] Granato D, de Araújo Calado VÔM, Jarvis B (2014) Observations on the use of statistical methods in Food Science and Technology. Food Res Int 55:137–149. 10.1016/J.FOODRES.2013.10.024

[27] Penteado AL, Eblen BS, Miller AJ (2004) Evidence of *Salmonella* Internalization into Fresh Mangos during Simulated Postharvest Insect Disinfestation Procedures. J Food Prot 67(1):181–184. 10.4315/0362-028X-67.1.181 14717371 10.4315/0362-028x-67.1.181

[28] Jung J, Friedrich LM, Danyluk MD, Schaffner DW (2017) Quantification of Transfer of *Salmonella* from Citrus Fruits to Peel, Edible Portion, and Gloved Hands during Hand Peeling. J Food Prot 80(6):933–939. 10.4315/0362-028X.JFP-16-423 28463082 10.4315/0362-028X.JFP-16-423

[29] Rezende ACB, Crucello J, Moreira RC, Silva BS, Sant’Ana AS (2016) Incidence and growth of *Salmonella enterica* on the peel and pulp of avocado (*Persea americana*) and custard apple (*Annona squamosa*). Int J Food Microbiol 235:10–16. 10.1016/J.IJFOODMICRO.2016.06.034 27393884 10.1016/j.ijfoodmicro.2016.06.034

[30] International Commission on Microbiological Specifications for Foods of the International Union of Biological Societies (ICMSF) (1996) Micro-Organisms in Foods: Microbiological Specifications of Food Pathogens. Blackie Academic & Professional, London, UK

[31] Food and Drug Administration (FDA) (2012) Bad Bug Book: Foodborne Pathogenic Microorganisms and Natural Toxins Handbook. 2nd ed

[32] Abadias M, Usall J, Anguera M, Solsona C, Viñas I (2008) Microbiological quality of fresh, minimally-processed fruit and vegetables, and sprouts from retail establishments. Int J Food Microbiol123(1–2):121–129. 10.1016/J.IJFOODMICRO.2007.12.013 10.1016/j.ijfoodmicro.2007.12.013 18237811

[33] Caponigro V, Ventura M, Chiancone I, Amato L, Parente E, Piro F (2010) Variation of microbial load and visual quality of ready-to-eat salads by vegetable type, season, processor and retailer. Food Microbiol 27(8):1071–1077. 10.1016/J.FM.2010.07.011 20832687 10.1016/j.fm.2010.07.011

[34] Russell NJ (2002) Bacterial membranes: the effects of chill storage and food processing. An overview. Int J Food Microbiol 79(1–2):27–34. 10.1016/S0168-1605(02)00176-9 12382682 10.1016/s0168-1605(02)00176-9

[35] Beuchat LR (2002) Ecological factors influencing survival and growth of human pathogens on raw fruits and vegetables. Microbes Infect 4(4):413–423. 10.1016/S1286-4579(02)01555-1 11932192 10.1016/s1286-4579(02)01555-1

[36] Morey A, Singh M (2012) Low-temperature survival of *Salmonella* spp. in a model food system with natural microflora. Foodborne Pathog Dis 9(3):218–223. 10.1089/FPD.2011.1016 22217013 10.1089/fpd.2011.1016

[37] De Lucena EMP, De Assis JS, Alves RE, Da Silva VCM, Enéas Filho J (2007) Alterações físicas e químicas durante o desenvolvimento de mangas Tommy Atkins no vale de São Francisco, Petrolina-PE. Rev Bras Frutic 29(1):96–101. 10.1590/S0100-29452007000100021

[38] Queiroga V, de Gomes P, Melo JP et al (2023) BA, Manga (Manguifera Indica, L. Cv. Tommy Atkins). 1a Edição. Albuquerque, Esther Maria Barros. Accessed August 19, 2025. https://www.researchgate.net/profile/Nouglas-Mendes2/publication/375837910_MANGA_Mangifera_indica_L_cv_Tommy_Atkins/links/655f305bce88b8703107c53d/MANGA-Mangifera-indica-L-cv-Tommy-Atkins.pdf

[39] Beuchat LR, Mann DA (2008) Survival and Growth of Acid-Adapted and Unadapted Salmonella in and on Raw Tomatoes as Affected by Variety, Stage of Ripeness, and Storage Temperature. J Food Prot 71(8):1572–1579. 10.4315/0362-028X-71.8.1572 18724750 10.4315/0362-028x-71.8.1572

[40] Strawn LK, Danyluk MD (2010a) Fate of *Escherichia coli* O157: H7 and *Salmonella* on Fresh and Frozen Cut Pineapples. J Food Prot 73(3):418–424. 10.4315/0362-028X-73.3.418 20202325 10.4315/0362-028x-73.3.418

[41] Jay JM, Loesner M, Golden DA (2005) Modern Food Microbiology, 7th edn. Chapman and Hall, New York, USA

[42] John W, Foster MP, Spector (1995) How *Salmonella* survive against the odds. Annual Review Microbiology 49:145–174. Accessed August 14, 2025. https://d1wqtxts1xzle7.cloudfront.net/66019811/How_Salmonella_survive_against_the_odds20210315-17545-42eg5v 10.1146/annurev.mi.49.100195.001045 8561457

[43] Strawn LK, Danyluk MD (2010b) Fate of *Escherichia coli* O157:H7 and *Salmonella* spp. on fresh and frozen cut mangoes and papayas. Int J Food Microbiol 138(1–2):78–84. 10.1016/J.IJFOODMICRO.2009.12.002 20022397 10.1016/j.ijfoodmicro.2009.12.002

[44] Medlicott AP (1990) Ripening of Mangos Following Low-temperature Storage. Am Soc Hortic Sci 115(3):430. https://journals.ashs.org Accessed August 14, 2025

[45] Hatton TT (1990) Reduction of chilling injury with temperature manipulation. Published online 1990269-280. Accessed August 14, 2025. https://books.google.com/books/about/Chilling_Injury_of_Horticultural_Crops.html?hl=pt-BR&id=uosKSz7urC8C

[46] Baranyi J, Robinson TP, Kaloti A, Mackey BM (1995) Predicting growth of *Brochothrix thermosphacta* at changing temperature. Int J Food Microbiol 27(1):61–75. 10.1016/0168-1605(94)00154-X 8527329 10.1016/0168-1605(94)00154-x

[47] Ukuku DO, Huang L, Sommers C (2015) Efficacy of Sanitizer Treatments on Survival and Growth Parameters of *Escherichia coli* O157:H7, *Salmonella*, and *Listeria monocytogenes* on Fresh-Cut Pieces of Cantaloupe during Storage. J Food Prot 78(7):1288–1295. 10.4315/0362-028X.JFP-14-233 26197279 10.4315/0362-028X.JFP-14-233

[48] Ukuku DO, Fett WF (2006) Effects of Cell Surface Charge and Hydrophobicity on Attachment of 16 *Salmonella* Serovars to Cantaloupe Rind and Decontamination with Sanitizers. J Food Prot 69(8):1835–1843. 10.4315/0362-028X-69.8.1835 16924907 10.4315/0362-028x-69.8.1835

[49] Wang X, Topalcengiz Z, Danyluk MD (2024) Assessing the efficacy of sanitizer sprays during brush or polyvinyl chloride (PVC) roller treatment to reduce *Salmonella* populations on whole mangoes. Food Res Int 191:114590. 10.1016/J.FOODRES.2024.114590 39059891 10.1016/j.foodres.2024.114590

[50] Balali GI, Yar DD, Afua Dela VG, Adjei-Kusi P (2020) Microbial Contamination, an Increasing Threat to the Consumption of Fresh Fruits and Vegetables in Today’s World. Int J Microbiol 13029295. 10.1155/2020/3029295 10.1155/2020/3029295 PMC7269610 32565813

[51] Sivapalasingam S, Friedman CR, Cohen L, Tauxe RV (2004) Fresh Produce: A Growing Cause of Outbreaks of Foodborne Illness in the United States, 1973 through 1997. J Food Prot 67(10):2342–2353. 10.4315/0362-028X-67.10.2342 15508656 10.4315/0362-028x-67.10.2342

[52] Buchanan RL, Edelson SG, Miller RL, Sapers GM (1999) Contamination of Intact Apples after Immersion in an Aqueous Environment Containing *Escherichia coli* O157:H7. J Food Prot 62(5):444–450. 10.4315/0362-028X-62.5.444 10340662 10.4315/0362-028x-62.5.444

[53] Zhuang RY, Beuchat LR, Angulo FJ (1995) Fate of *Salmonella* montevideo on and in raw tomatoes as affected by temperature and treatment with chlorine. Appl Environ Microbiol 61(6):2127–2131. 10.1128/AEM.61.6.2127-2131.1995 7793934 10.1128/aem.61.6.2127-2131.1995 PMC167485

[54] Gkana E, Lianou A, Nychas GJE (2016) Transfer of *Salmonella enterica* Serovar Typhimurium from Beef to Tomato through Kitchen Equipment and the Efficacy of Intermediate Decontamination Procedures. J Food Prot 79(7):1252–1258. 10.4315/0362-028X.JFP-15-531 27357047 10.4315/0362-028X.JFP-15-531

[55] Lin CM, Wei CI (1997) Transfer of *Salmonella* montevideo onto the Interior Surfaces of Tomatoes by Cutting. J Food Prot 60(7):858–862. 10.4315/0362-028X-60.7.858 31026878 10.4315/0362-028X-60.7.858

[56] Wang H, Ryser ET (2016) Quantitative transfer of *Salmonella* Typhimurium LT2 during mechanical slicing of tomatoes as impacted by multiple processing variables. Int J Food Microbiol 234:76–82. 10.1016/J.IJFOODMICRO.2016.06.035 27382959 10.1016/j.ijfoodmicro.2016.06.035

[57] Bhattacharjee P, Warang O, Das S, Das S, Kashmir I (2022) Impact of Climate Change on Fruit Crops-A Review Article History. Curr World Environ 4929(2):319–330. 10.12944/CWE.17.2.4

[58] Lopes MM, de Lucena A, de Silveira HH et al (2021) MRS da, The use of electrolyzed water as a disinfectant for fresh cut mango. Sci Hortic 287:110227. 10.1016/J.SCIENTA.2021.110227

